# *STUB1*/CHIP mutations cause Gordon Holmes syndrome as part of a widespread multisystemic neurodegeneration: evidence from four novel mutations

**DOI:** 10.1186/s13023-017-0580-x

**Published:** 2017-02-13

**Authors:** Stefanie Nicole Hayer, Tine Deconinck, Benjamin Bender, Katrien Smets, Stephan Züchner, Selina Reich, Ludger Schöls, Rebecca Schüle, Peter De Jonghe, Jonathan Baets, Matthis Synofzik

**Affiliations:** 1 Department of Neurodegenerative Diseases, Hertie-Institute for Clinical Brain Research & Center of Neurology, University of Tuebingen, Hoppe-Seyler-Str. 3, 72076 Tuebingen, Germany; 2 German Center for Neurodegenerative Diseases (DZNE), University of Tuebingen, Tuebingen, Germany; 3 Neurogenetics Group, Department of Molecular Genetics, VIB, Antwerp, Belgium; 4 Laboratories of Neurogenetics and Ultrastructural Neuropathology, Institute Born-Bunge, University of Antwerp, Antwerp, Belgium; 5 Department of Diagnostic and Interventional Neuroradiology, University Hospital Tuebingen, Tuebingen, Germany; 6 Department of Neurology, Antwerp University Hospital, Antwerp, Belgium; 7 Dr. John T. Macdonald Foundation, Department of Human Genetics, Miami, USA; 8 John P. Hussman Institute for Human Genomics, University of Miami, Miller School of Medicine, Miami, USA

**Keywords:** Neurodegeneration, Neurodegenerative disease, CHIP, Gordon Holmes syndrome, Ataxia, Recessive ataxia, Spastic ataxia, Early onset ataxia, Dementia, Early-onset dementia, Hypogonadism, Magnetic resonance imaging

## Abstract

**Background:**

CHIP, the protein encoded by *STUB1*, is a central component of cellular protein homeostasis and interacts with several key proteins involved in the pathogenesis of manifold neurodegenerative diseases. This gives rise to the hypothesis that mutations in *STUB1* might cause a far more multisystemic neurodegenerative phenotype than the previously reported cerebellar ataxia syndrome.

**Methods:**

Whole exome sequencing data-sets from *n* = 87 index subjects of two ataxia cohorts were screened for individuals with *STUB1* mutations. In-depth phenotyping by clinical evaluation and neuroimaging was performed in mutation carriers.

**Results:**

We identified four novel *STUB1* mutations in three affected subjects from two index families (frequency 2/87 = 2.3%). All three subjects presented with a severe multisystemic phenotype including severe dementia, spastic tetraparesis, epilepsy, and autonomic dysfunction in addition to cerebellar ataxia, plus hypogonadism in one index patient. Diffusion tensor imaging revealed degeneration of manifold supra- and infratentorial tracts.

**Conclusions:**

Our findings provide clinical and imaging support for the notion that CHIP is a crucial converging point of manifold neurodegenerative processes, corresponding with its universal biological function in neurodegeneration. Further, our data reveal the second *STUB1* family with ataxia plus hypogonadism reported so far, demonstrating that Gordon Holmes syndrome is indeed a recurrent manifestation of *STUB1*. However, it does not present in isolation, but as part of a broad multisystemic neurodegenerative process. This supports the notion that *STUB1* disease should be conceptualized not by historical or clinical syndromic names, but as a variable multisystemic disease defined by disturbed function of the underlying *STUB1* gene, which translates into a multidimensional gradual spectrum of variably associated clinical signs and symptoms.

**Electronic supplementary material:**

The online version of this article (doi:10.1186/s13023-017-0580-x) contains supplementary material, which is available to authorized users.

## Background

Mutations in *STUB1*, the gene encoding the protein CHIP (C-terminus of HSC70-interacting Protein), were recently identified as a cause of autosomal recessive cerebellar ataxia (ARCA) in several families [1–5]. Most of the reported individuals with *STUB1* mutations had a relatively circumscribed phenotype, mainly consisting of an ataxia syndrome with involvement of only one or two additional neurologic systems. For example, in one index patient hypogonadotropic hypogonadism was identified concomitant to ataxia, leading to the notion that *STUB1* is a cause of Gordon Holmes syndrome [5, 6]. Yet it remains unclear whether hypogonadism is a systematic part of the phenotypic spectrum of *STUB1* and not just a coincidental finding in a single family [5].

The current notion of *STUB1* as causing a relatively circumscribed ‘ataxia plus phenotype’ is remarkable, since CHIP, the protein encoded by *STUB1*, is a key component of general cellular protein homeostasis [7, 8] and interacts with several proteins involved in the pathogenesis of various neurodegenerative diseases and system degenerations, including Tau, α-Synuclein, Parkin2, LRRK2, Ataxin1, Ataxin3, and ATCAY (for references and overview, see Figure Additional file 1). Accordingly, it is to be expected that in patients with *STUB1* mutations, disruption of CHIP function might lead to far more extensive neurodegeneration than the previously reported cerebellar ataxia syndrome. Specifically, given the central role of CHIP in protein homeostasis and its interactions with many neurodegenerative proteins, we hypothesized that mutant *STUB1* should lead to damage of almost all brain systems.

Here we report the clinical, genetic and imaging findings from three novel *STUB1* patients and four novel *STUB1* mutations. We demonstrate that mutant *STUB1* leads to severe multisystemic neurodegeneration affecting almost all brain tracts and, correspondingly, presenting with a broad multisystemic phenotype including severe dementia, spastic tetraparesis, epilepsy, and autonomic dysfunction in addition to cerebellar ataxia. These clinical and imaging findings correspond with the broad protein interactome of CHIP with other neurodegenerative disease proteins. Moreover, our data provide the first confirmation from an independent family showing that hypogonadotropic hypogonadism is indeed a recurrent part of the phenotypic spectrum of *STUB1* mutations, rendering them an important cause of Gordon Holmes syndrome; yet not in isolation, but as part of a broad multisystemic neurodegenerative process. This indicates that *STUB1* disease should not be conceptualized by distinct syndromic names, but as a variable multisystemic disease defined by disturbed function of the underlying *STUB1* gene, which translates into a multidimensional gradual spectrum of variably associated signs and symptoms.

## Methods

### Genetic sequencing

Whole exome sequencing (WES) data-sets from *n* = 87 index subjects of two ataxia cohorts (*n* = 35 from Tuebingen, Germany; exomes generated from 2014 and 2015; and *n* = 52 from Antwerp, Belgium exomes generated from 2012 until 2015) were screened for individuals with biallelic *STUB1* mutations. Cohorts comprised subjects with early-onset degenerative ataxia compatible with autosomal inheritance (i.e. progressive ataxia with onset <40 years with ataxia in no more than one generation), negative for trinucleotide repeat expansions causing Friedreich’s ataxia and spinocerebellar ataxia type 1, 2, 3, 6, 7, and 17. WES was performed using the SureSelect Human All Exon 50 Mb kit (Agilent, Santa Clara, CA, USA) for in-solution enrichment and the Hiseq2000 instrument (Illumina, San Diego, CA, USA) as described before [9]. All data were then annotated and imported into the GENESIS (gem.app) platform, a web-based tool for next generation sequencing data analysis (http://thegenesisprojectfoundation.org/) [10, 11]. Variants were filtered for (i) non-synonymous homozygous or compound heterozygous mutations in *STUB1* that were (ii) absent or extremely rare (minor allele frequency <0.5%) in the public databases dbSNP137, NHLBI ESP6500, 1000Genomes project, and ExAc (60706 exomes; Exome Aggregation Consortium; Cambridge, MA http://exac.broadinstitute.org) as well as in GENESIS (<11 heterozygous or homozygous alleles in 5996 subjects in the GENESIS database), and showed an at least (iii) moderate conservation (PhastCons score [100 vertebrate genomes] >0.5 AND phyloP [100 vertebrate genomes] >1.5) and (iv) moderate genotype quality (quality filter [QUAL] >35 and genotype quality GQ > 50).

The data collection was approved by the Ethics Committees of the University Hospital Antwerp and the University Hospital Tuebingen (598/2011BO1).

### In-depth phenotyping

All *STUB1* subjects were examined by an experienced neurologist (Tuebingen patients: M.S.; Antwerp patients: J.B.) according to a standardized clinical assessment protocol covering all neurological systems, including scales to capture cognition, spasticity, ataxia, and overall handicap (Spinocerebellar degeneration functional score [SDFS]). This SDFS evaluates the disability stage from 0 to 7 (0: no functional handicap; 1: no functional handicap but signs at examination; 2: mild, able to run, walking unlimited; 3: moderate, unable to run, limited walking without help; 4: severe, walking with one stick; 5: walking with two sticks; 6: unable to walk, requiring wheelchair; 7: confined to the bed) [12].

### Magnetic resonance imaging (MRI)

In addition to clinical routine cerebral imaging, performed in all *STUB1* subjects, detailed diffusion tensor imaging (DTI) was performed on a 3 Tesla scanner (Skyra, Siemens Healthcare, Erlangen, Germany) with a 32 channel head coil in one *STUB1* subject. DTI data was acquired with 64 diffusion directions (b = 1000 s/mm^2^) and one b0 image with an isotropic resolution of 2 mm and coverage of the whole head. The patient and nine age and gender matched healthy controls (mean age 32.9 years, range 27–38 years) were examined at the same scanner with the same DTI protocol. Data were processed with Tract-Based Spatial Statistics (TBSS) [13]. TBSS projects all subjects’ fractional anisotropy (FA) data onto a mean FA tract skeleton which represents the centres of all tracts common to the group. Usually, such maps are analysed voxel-wise for significant differences between groups. However, given the group size of *n* = 1 for the patient, a voxel-wise comparison would lead to many misleading false positive and false negative results. Therefore, we here compared the FA of whole tracts defined by the 48 labelled white matter tracts of the ICBM-DTI-81 atlas [14] provided in FSL (FMRIB Sofware Library, available at http://fsl.fmrib.ox.ac.uk/fsl/fslwiki/) between the patient and the control group. All labels that covered at least 600 voxels of the skeleton were evaluated with a *t*-test for significant between-group differences between the healthy control group and the *STUB1* patient. To correct for multiple comparison the Bonferroni method was used and the significance level was set at α < 0.0011.

For methods of the western blot analysis, see Additional file 2. For methods of the CHIP protein-protein-network analysis, see Additional file 1.

## Results

### Genetic findings

**Fig. 1:**
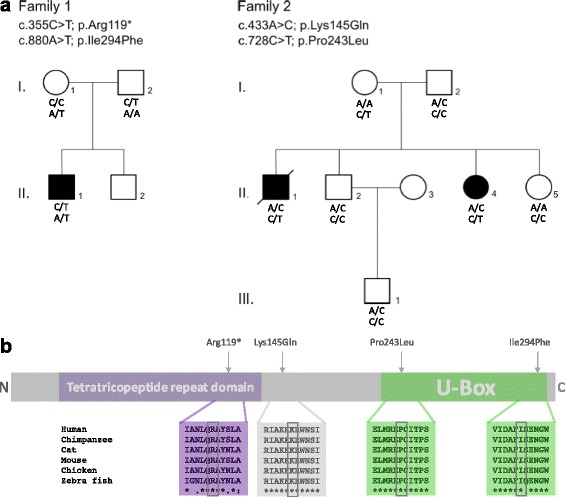
Pedigrees of *STUB1* families and domain location of the four novel *STUB1* mutations. **a** Compound heterozygous *STUB1* mutations and pedigrees of the two reported families. In family 1, one affected individual (II.1) carried the compound heterozygeous mutations p.Arg119* and p.Ile294Phe. In family 2, two affected siblings (II.1 and II.4) both carried the mutations p.Lys145Gln and p.Pro243Leu. **b** Schematic representation of CHIP, the protein encoded by *STUB1,* with the highly conserved N-terminal tetratricopeptide repeat and C-terminal U-box domain. Of the four novel mutations, two are located in the U-Box domain (p.Pro243Leu and p.Ile294Phe), one in between the conserved domains (Lys145Gln) and one is predicted to locate to the tetratricopeptide repeat domain (p.Arg119*), which, however, most probably leads to nonsense-mediated decay on RNA level

WES revealed four *STUB1* mutations (one nonsense, three missense), all of them not previously linked to human disease, in subjects from two different index families (2/87 = 2.3% frequency in total cohort) from Central Europe (family 1: German origin; family 2 Belgian origin). Subject II.1 of family 1 carried the variant c.355C > T in Exon 2, which is predicted to result in a stop codon at position 119 (p.Arg119*), most probably leading to nonsense-mediated mRNA decay due to the premature stop codon. The second variant, c.880A > T in Exon 7, leads to an amino acid exchange from isoleucine to phenylalanine at position 294 (p.Ile294Phe) (Fig. 1a), affecting the highly conserved U-Box domain of the protein (Fig. 1b). Subjects II.1 and II.4 of family two carried the *STUB1* missense variants, c.433A > C in Exon 3, p.Lys145Gln, located outside the tetratricopeptide repeat sequence and the U-Box domain (Fig. 1b); and c.728C > T in Exon 6, p.Pro243Leu (Fig. 1a) which affects the U-Box domain of the protein. All four variants were absent or very rare in in the Exome Variant Server (EVS) and in-house databases (Table 1).

**Table 1:** Summary of the novel *STUB1* mutations

Sybject	Family 1, II.1		Family 2, II.1 + II.4	
Phenotype	Dementia, upper motor neuron damage, hypogonadism, ataxia, epilepsy		Dementia, upper motor neuron damage, epilepsy, ataxia	
Genomic position	Chr16:732457	Chr16:731347	Chr16:731512	Chr16:732223
cDNA change	c.880A > T	c.355C > T	c.433A > C	c.728C > T
Protein change	p.Ile294Phe	p.Arg119*	p.Lys145Gln	p.Pro243Leu
GVS Function	missense	nonsense	missense	missense
PhyloP 100	4.5	1.6	7.07	5.85
PolyPhen2 (div)	probably damaging	NA	possibly damaging	probably damaging
SIFT	D	NA	D	D
Mutation Taster	D	D	D	D
ExAc/EVS/1000G	0	0	0.001/0.001/0.001	0/NA/NA
GENESIS allele counts	1	1	6 (het)	1

Overview of the mutations including phenotypic features, rating by the mutation prediction softwares PhyloP, PolyPhen2, SIFT, and Mutation Taster and a summary of the allele frequency in the databases ExAc/EVS/1000G MAF and GENESIS. Legend: *NA* not applicable. *ExAc* Exome Aggregation Consortium, *EVS* Exome Variant Server, 1000G *MAF* 1000 Genomes minor allele frequency, *het* heterozygous, *GVS* Genome Variant Server

All three missense mutations were predicted to be damaging by at least three different *in silico* prediction tools (Table 1). All mutations were confirmed by Sanger sequencing. Biallelic localization of the respective *STUB1* variants in *trans* was confirmed in both families by testing for a heterozygous state of the respective variant in the parents. In line with the fact that affected patients of both *STUB1* families carried at least one missense *STUB1* variant (rather than two truncating variants), no truncation of the CHIP protein was observed (Additional file 2).

### Clinical findings

A summarized overview of clinical symptoms in all three affected subjects is provided in Table 2 (for detailed case vignettes, see Additional file 3). Subject II.1, family 1, presented with slightly delayed early motor development, generalized tonic-clonic seizures (years 1–2 of life) and undescended testes (surgery at age 7). He did not show ataxia symptoms until age 12. Also subject II. 4 of family 2 did not present with ataxia as initial symptom, but with cataracts requiring surgery at the age of 11. Ataxia did not start before age 20. Only in subject II.1, *STUB1* disease started with ataxia as initial symptom (age 12 years), followed by spasticity starting at the age of 20 and cognitive deficits starting at 23 years. All three subjects developed severe dementia with predominantly frontal-executive dysfunction and progressive loss of language in the first three decades of life, leading to mutism in 2/3 cases before the age of 40. Likewise, all three subjects developed severe pyramidal tract damage to arms and legs, including incapacitating tetraspasticity. Extrapyramidal hyperkinetic movement disorders included choreo-athetotic movements in 2/3 subjects and dystonia in 1/3 subjects. Gait disturbances were quickly progressive in all three subjects, leading to wheelchair-dependency 6, 9, and 15 years after respective onset of gait difficulties. All three subjects developed severe dysphagia, starting between age 20 to age 35, and necessitating gastric tube feeding in both subjects from family 2 at the age of 36 and 43, respectively. This severe multisystemic neurodegenerative disease led to complete care dependency in all three subjects before aged 40, and premature death at the age of 40 in one of them.

**Table 2:** Summary of clinical, imaging, and laboratory data of the *STUB1* patients

Family	1	2	2
Subject	II.1	II.1	II.4
STUB1 mutation	c.355C > T p.Arg119* + c.880A > T p.Il294Phe	c. 433A > C p.Lys145Gln + c.728C > T p.Pro243Leu	c.433A > C p.Lys145Gln + c.728C > T p.Pro243Leu
Gender	M	M	F
Age at last investigation	34y	35y (patient died aged 40)	45y
First symptom, age of onset	epilepsy, 2y	ataxia, age 12y	cataract surgery left eye, 11y
Ataxia, age of onset	12y	12y	20y
Tendon reflexes	increased in UE/LE	increased in UE/LE	increased in UE/LE
Spacticity	+++ in UE and LE	+ in UE and LE	+ in UE and LE
Babinski’s sign	+ bilateral	+ bilateral	+ bilateral
Ankle clonus	-	+ bilateral	+ bilateral
Urge incontinence	+	+	+ (40y)
Parkinsonism	hypomimia	-	-
Hyperkinetic movements (dystonia/athetosis)	focal dystonia upper limb	intermittend ballistic athetotic movements	intermittend ballistic athetotic movements
Epilepsy	GTCS in early childhood	GTCS (onset 35y)	GTCS? (onset 42y)
Muscle atrophy	distal UE/LE, possibly secondary to disuse	generalized UE/LE atrophy secondary to disuse	distal UE/LE, possibly secondary to disuse
Sense of vibration	cannot be tested reliability due to dementia	cannot be tested reliability due to dementia	cannot be tested reliability due to dementia
Cognitive impainment	severe	severe, mutism, PEG at 36y	severe, mutism, PEG at 43y
Neuropsychology	not testable anymore due to too severe cognitive deficits	not testable anymore; TIQ 85 (WAIS) at 32y	not testable anymore; MMSE 29/30 at 24y, work as secretary in early 20ies
SDFS	6	6	6
SARA	36	40	40
SPRS	36	34	40
Nerve conduction studies	sural and tibial nerve normal	sural and tibial nerve normal	sural and tibial nerve normal
Motor evoked potentials	n/a	normal	normal (SSEP’s and BAEP also normal)
Cerebral imaging	cerebellar, mesencephalic and parieto-occipital cortical atrophy	cerebellar atrophy	severe cerebellar atrophy, vermis and hemispheric, brainstem normal (33y)
Hypogonadism	+	secondary sex characteristics present	secondary sex characteristics present
Hormones	Testosteron 5,2 nmol/I; LH 0,8 IU/I; FSH 0,8 IU/I	normal (36y)	n/a
Testicular volume (sonography)	right testicle: 4.2 ml left testicle: 3.9 ml	n/a	not applicable

Legend: *M* male, *F* female, *y* years, *n/a* not applicable, *UE* upper extremity, *LE* lower extremity, *GTCS* generalized tonic-clonic seizure, *TIQ* total intelligence quotient, *WAIS* Wechsler Adult Intelligence Scale, *MMSE* Mini Mental State Examination, *SDFS* Spinocerebellar Degeneration Functional Score. This score was used to evaluate the disability stage from 1 to 7 (0: no functional handicap; 1: no functional handicap but signs at examination; 2: mild, able to run, walking unlimited; 3: moderate, unable to run, limited walking without help; 4: severe, walking with one stick; 5: walking with two sticks; 6: unable to walk, requiring wheelchair; 7: confined to the bed). SARA, Scale for the Assessment and Rating of Ataxia, reaching from 0 to 40, with higher scores indicating more severe ataxia [17]; scores <3 points are considered unspecific. SPRS, Spastic Paraplegia Rating Scale, reaching from 0 to 52, with higher scores indicating more severe spastic paraplegia [18] (please note, however, that several items of the SPRS scale increase also with more severe ataxia); SSEP, somatosensory evoked potential; BAEP, brainstem auditory evoked potentials; LH, luteinizing hormone; FSH, follicle-stimulating hormone

### Neuroimaging

**Fig. 2:**
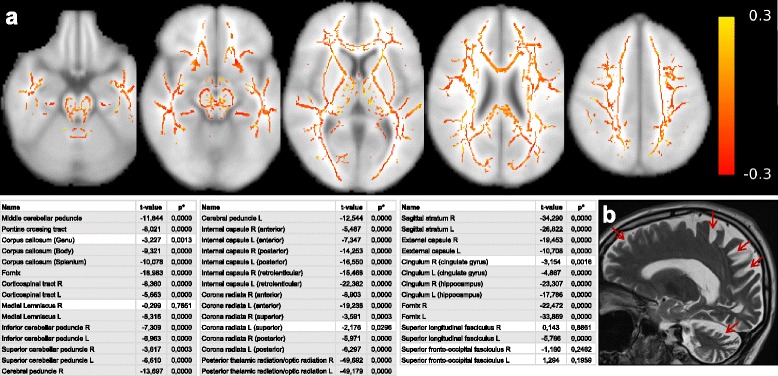
MR imaging features of an individual with *STUB1*/CHIP mutation. **a** Top: illustration of the FA differences between patient II.1, family 1 versus the healthy control group, overlaid onto a standard brain available in FSL. The mean FA skeleton was calculated voxelwise over all 9 control subjects, and then subtracted from the FA skeleton of the *STUB1* patient. Red color encodes a negative difference, i.e. a decreased FA in the *STUB1* subject compared to the mean FA of the controls. *Yellow color* encodes an increased FA in the *STUB1* subject compared to the mean FA of the controls. Individual FA can theoretically range from 0 to 1, in vivo FA usually ranges between 0.05 in GM and 0.9 in large WM tracts. Over the whole skeleton negative values are much more common, in line with the statistical evaluation of whole fiber tracts: *Bottom*: corresponding list of all brain tracts, and the results of a *t*-test of the voxels of each tract comparing the *STUB1* subject with the healthy control group. Tracts with *gray background* are statistically significant. **b** Sagittal T2 MRI showing marked cerebellar degeneration and global cerebral atrophy with an emphasis on the parietal and occipital lobes in subject II.1 of family 1 (arrows). FA, fractional anisotropy; FSL, FMRIB Sofware Library; GM, gray matter; WM, white matter

Routine imaging (cerebral magnetic resonance imaging [cMRI] in two subjects, cerebral computer tomography [cCT] in one subject) showed cerebellar atrophy in all three subjects and, in addition, mesencephalic and parieto-occipital cortical atrophy in subject II.1, family 1. To reveal the atrophy pattern of different neural tracts in more detail, diffusion tension imaging (DTI) imaging was performed in subject II.1. It showed a widespread atrophy and globally reduced fractional anisotropy (FA) of literally all brain fiber tracts, from the corticospinal tract via the *corona radiata* to the superior, middle and inferior cerebellar peduncle (Fig. 2).

## Discussion

Our findings on four novel mutations in three novel *STUB1* subjects extend the genetic spectrum of *STUB1*, corroborating earlier findings that *STUB1* mutations both inside and outside of the tetratricopeptide repeat and U-Box domain of CHIP can lead to neurodegenerative disease [1]. The approximate frequency estimate of 2.3% confirms our previous data from an independent early-onset ataxia cohort that *STUB1* is a recurrent, but overall rare cause of early-onset ataxia (previous frequency estimate: 1.8%; [1]).

**Fig. 3:**
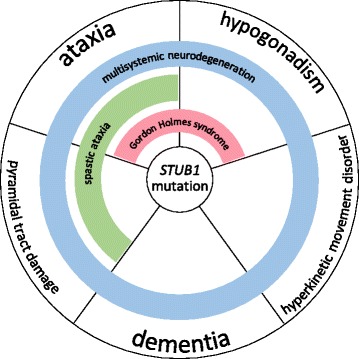
The unfolding phenotypic spectrum of *STUB1* disease. The clinical spectrum of *STUB1* mutations unfolds along five different neurological key features: ataxia, pyramidal tract damage, dementia, hypogonadism, and hyperkinetic movement disorders. Accordingly, each or several of these key features might be missing in single individuals with *STUB1* disease. Moreover, it illustrates that *STUB1* causes ataxia and hypogonadism (=Gordon Holmes syndrome) not in isolation, but as part of a continuous spectrum of *STUB1*-associated disease features. These features can be variably combined in *STUB1*-disease clusters, e.g. ataxia and hypogonadism (*red bar* ≙ Shi et al. [5]), ataxia with pyramidal tract damage (spastic ataxia; *green bar* ≙ Synofzik et al. [1]), or ataxia plus hypogonadism, pyramidal tract damage, dementia and hyperkinetic movement disorders, i.e. encompassing all *STUB1* disease features (*blue bar* ≙ current report)

More importantly, however, our findings provide evidence for a severe multisystemic neurodegenerative disease, corresponding with the universal protein function of CHIP in neurodegeneration (see Figs. 2, 3, and Figure Additional file 1). Specifically, in line with the universal biological role of CHIP as a crucial converging point of multiple pathways important for neuronal homeostasis (Additional file 1), the disease phenotype is not limited to an ataxia syndrome with minor involvement of one or two additional systems, but rather involves almost *all* brain tracts (Figs. 3 and 2). This is evidenced clinically by the fact that ataxia is only *one* feature of a broad multisystemic phenotype which includes severe dementia advancing to mutism, epilepsy, profound pyramidal tract damage including tetraspasticity, and extrapyramidal hyperkinetic movement disorders. In fact, ataxia was not even the first feature in the evolution of the disease in two out of the three affected subjects.

The notion of aberrant CHIP function leading not only to ataxia syndromes, but to a broad neurodegeneration, is further evidenced by DTI imaging. In line with the clinical findings, DTI demonstrates neurodegeneration of almost all brain fiber tracts, from the corticospinal tract via the *corona radiata* to the cerebellar peduncles. Taken together, our findings of widespread neurodegeneration affecting manifold brain systems provide clinical and imaging evidence that CHIP seems to be an important protein for cell survival in various neuronal cell types.

Finally, our data provide the first confirmation from an independent family showing that hypogonadotropic hypogonadism is indeed part of the phenotypic cluster of *STUB1* mutations. So far, only one *STUB1* family has been reported to include also hypogonadism as part of the clinical phenotype [5]. Our description of a second, independent case from a different ethnic background now confirms hypogonadism as part of the disease spectrum. It adds further support of *STUB1* as one important cause of Gordon Holmes syndrome [6], which shows a substantial genetic heterogeneity as it can also be caused by mutations in e.g. *PNPLA6* [9]. Importantly, we demonstrate here that *STUB1* causes the Gordon Holmes syndrome (early onset ataxia plus hypogonadism) not in isolation, but rather as part of a broad multisystemic neurodegenerative process (see Fig. 3). It thus resembles also other genes which cause Gordon Holmes syndrome as part of a multisystemic disease spectrum, e.g. PNPLA6 [9].

Correspondingly, in line with the reclassifications of other neurodegenerative diseases [15, 16], we suggest viewing *STUB1*-associated disease not in terms of syndromic names (e.g. “ataxia-dementia-hypogonadotropism syndrome” or “Gordon Holmes syndrome”); rather, it should be conceptualized as a fluid, complex, multisystemic neurodegenerative disease affecting various regions and/or systems of the nervous system (cerebellar, extrapyramidal, pyramidal, cortical, endocrine) defined by disturbed *STUB1* function that translates phenotypically into a multidimensional gradual spectrum of variably associated signs and symptoms (Fig. 3).

## Conclusion

Our findings provide clinical and imaging support for the notion that CHIP is a crucial converging point of widespread multisystemic neurodegenerative processes, thus corresponding with its universal biological function in neuronal homeostasis. Further, we show that Gordon Holmes syndrome presents as part of this widespread, variable multisystemic neurodegenerative process.

## Additional files

Additional file 1: Protein network analysis. (DOCX 1176 kb)
Additional file 2: Western blots of CHIP in mutation carriers. (DOCX 241 kb)
Additional file 3: Case vignettes. Detailed medical history and clinical examination data of the three *STUB1* patients. (DOCX 14 kb)

## Abbreviations

ARCA: Autosomal recessive cerebellar ataxia
ATCAY: Ataxia, cerebellar, Cayman type
BAEP: Brainstem auditory evoked potentials
CHIP: C-terminus of HSC70-interacting Protein
CT: Computer tomography
DTI: Diffusion tensor imaging
EVS: Exome Variant Server
ExAc: Exome Aggregation Consortium
F: Female
FA: Fractional anisotropy
FSH: Follicle-stimulating hormone
GM: Gray matter
GTCS: Generalized tonic-clonic seizure
GVS: Genome variant server
LE: Lower extremity
LH: Luteinizing hormone
M: Male
MAF: Minor allele frequency
MMSE: Mini Mental State Examination
MRI: Magnetic resonance imaging
SARA: Scale for the Assessment and Rating of Ataxia
SDFS: Spinocerebellar Degeneration Functional Score
SPRS: Spastic Paraplegia Rating Scale
SSEP: Somatosensory evoked potential
STUB1: STIP1 homology and U-box containing protein 1
TBSS: Tract-based spatial statistics
TIQ: Total intelligence quotient
UE: Upper extremity
WAIS: Wechsler Adult Intelligence Scale
WES: Whole exome sequencing
WM: White matter
