# Supplementary material for: STUB1/CHIP mutations cause Gordon Holmes syndrome as part of a widespread multisystemic neurodegeneration: evidence from four novel mutations
Source: Orphanet J Rare Dis. 2017 Feb 13;12:31. doi: 10.1186/s13023-017-0580-x (PMC5307643; doi:10.1186/s13023-017-0580-x)
Supplement: Additional file 1: — Protein network analysis. (DOCX 1176 kb) [file 13023_2017_580_MOESM1_ESM.docx]

**Additional file 1: Protein network analysis**

*Methods*

Protein interactions of *STUB1*/CHIP were created using the Biological General Repository for Interaction Datasets (BioGRID) [1]. We filtered for all interactions with known neurodegenerative markers with a minimum evidence of 3 (low throughput). A literature search on these interactions was performed to characterize the functional implications of CHIP mutation. Cross-species conserved domain alignment was carried out using Clustal Omega [2].

*Results*

The analysis revealed an interactome of CHIP comprising 251 proteins [1], among them several neurodegenerative marker proteins including Tau, α-Synuclein, Parkin2, LRRK2, Ataxin1, Ataxin3, and ATCAY [3–13] (see Figure Additional File 3). The large number of interactors reflects the function of CHIP as an E3 ligase in the ubiquitin proteasome system [14] and its importance in cellular protein homeostasis. In cooperation with heat shock proteins [14–16], CHIP is responsible for the ubiquitination and degradation of numerous proteins including several proteins involved in the pathogenesis of neurodegenerative diseases. For instance, CHIP ubiquitinates phosphorylated Tau (pTau), a hallmark protein in Alzheimer’s disease and several other forms of dementia. Ubiquitination of pTau by CHIP leads to its proteasomal degradation, and protects from pTau-induced cell death [7–10, 17, 18]. Similarly, CHIP promotes the degradation of α-Synuclein and LRRK2, two proteins involved in the pathogenesis of Parkinson’s disease [6, 12, 13]. In addition to its importance in protein homeostasis, CHIP is also involved in the regulation of enzyme activity; it enhances the activity level of Ataxin3, a deubiquitinating enzyme implicated in the pathology of spinocerebellar ataxia type 3 (SCA3), that cooperates with CHIP to effectively degrade proteins via the ubiquitin proteasome pathway [19–22]. Further, CHIP increases the ability of the E3 ligase Parkin to ubiquitinate its target proteins [23]. Taken together, the analysis of the CHIP network indicates that CHIP is a crucial converging point of multiple pathways important for neuronal homeostasis.

**Figure Legend Additional File3: STUB1/CHIP interaction partners**. *STUB1*/CHIP interactions with several known neurodegenerative markers, including proteins involved in the pathology of Ataxia (ATX1, ATX3, ATCAY), Parkinson’s disease (LRRK2, PARK2, SNCA), Alzheimer’s disease (Tau), and other types of dementia (MAPT, SNCA). Greater node size represents increased connectivity; thicker edge (line) sizes represent increased evidence supporting the association. APP, amyloid beta precursor protein; ATCAY, ataxia, cerebellar, Cayman type; ATX1, ataxin 1; ATX3, ataxin 3; LRRK2, leucine-rich repeat kinase 2; MAPT, microtubule-associated protein tau; PARK2, parkin RBR E3 ubiquitin protein ligase; PARK7, parkinson protein 7; SNCA, synuclein, alpha.

References

1. Stark C, Breitkreutz B-J, Reguly T, Boucher L, Breitkreutz A, Tyers M: **BioGRID: a general repository for interaction datasets.** *Nucleic Acids Res* 2006, **34**(Database issue):D535-9.

2. Sievers F, Wilm A, Dineen D, Gibson TJ, Karplus K, Li W, Lopez R, McWilliam H, Remmert M, Söding J, Thompson JD, Higgins DG: **Fast, scalable generation of high-quality protein multiple sequence alignments using Clustal Omega.** *Mol Syst Biol* 2011, **7**:539.

3. Grelle G, Kostka S, Otto A, Kersten B, Genser KF, Müller E-C, Wälter S, Böddrich A, Stelzl U, Hänig C, Volkmer-Engert R, Landgraf C, Alberti S, Höhfeld J, Strödicke M, Wanker EE: **Identification of VCP/p97, carboxyl terminus of Hsp70-interacting protein (CHIP), and amphiphysin II interaction partners using membrane-based human proteome arrays.** *Mol Cell Proteomics* 2006, **5**:234–44.

4. Tetzlaff JE, Putcha P, Outeiro TF, Ivanov A, Berezovska O, Hyman BT, McLean PJ: **CHIP targets toxic alpha-Synuclein oligomers for degradation.** *J Biol Chem* 2008, **283**:17962–8.

5. Kalia L V, Kalia SK, Chau H, Lozano AM, Hyman BT, McLean PJ: **Ubiquitinylation of α-synuclein by carboxyl terminus Hsp70-interacting protein (CHIP) is regulated by Bcl-2-associated athanogene 5 (BAG5).** *PLoS One* 2011, **6**:e14695.

6. Dimant H, Zhu L, Kibuuka LN, Fan Z, Hyman BT, McLean PJ: **Direct visualization of CHIP-mediated degradation of alpha-synuclein in vivo: implications for PD therapeutics.** *PLoS One* 2014, **9**:e92098.

7. Dickey CA, Koren J, Zhang Y-J, Xu Y-F, Jinwal UK, Birnbaum MJ, Monks B, Sun M, Cheng JQ, Patterson C, Bailey RM, Dunmore J, Soresh S, Leon C, Morgan D, Petrucelli L: **Akt and CHIP coregulate tau degradation through coordinated interactions.** *Proc Natl Acad Sci U S A* 2008, **105**:3622–7.

8. Shimura H, Schwartz D, Gygi SP, Kosik KS: **CHIP-Hsc70 complex ubiquitinates phosphorylated tau and enhances cell survival.** *J Biol Chem* 2004, **279**:4869–76.

9. Petrucelli L, Dickson D, Kehoe K, Taylor J, Snyder H, Grover A, De Lucia M, McGowan E, Lewis J, Prihar G, Kim J, Dillmann WH, Browne SE, Hall A, Voellmy R, Tsuboi Y, Dawson TM, Wolozin B, Hardy J, Hutton M: **CHIP and Hsp70 regulate tau ubiquitination, degradation and aggregation.** *Hum Mol Genet* 2004, **13**:703–14.

10. Sahara N, Murayama M, Mizoroki T, Urushitani M, Imai Y, Takahashi R, Murata S, Tanaka K, Takashima A: **In vivo evidence of CHIP up-regulation attenuating tau aggregation.** *J Neurochem* 2005, **94**:1254–63.

11. Al-Ramahi I, Lam YC, Chen H-K, de Gouyon B, Zhang M, Pérez AM, Branco J, de Haro M, Patterson C, Zoghbi HY, Botas J: **CHIP protects from the neurotoxicity of expanded and wild-type ataxin-1 and promotes their ubiquitination and degradation.** *J Biol Chem* 2006, **281**:26714–24.

12. Ko HS, Bailey R, Smith WW, Liu Z, Shin J-H, Lee Y-I, Zhang Y-J, Jiang H, Ross CA, Moore DJ, Patterson C, Petrucelli L, Dawson TM, Dawson VL: **CHIP regulates leucine-rich repeat kinase-2 ubiquitination, degradation, and toxicity.** *Proc Natl Acad Sci U S A* 2009, **106**:2897–902.

13. Ding X, Goldberg MS: **Regulation of LRRK2 stability by the E3 ubiquitin ligase CHIP.** *PLoS One* 2009, **4**:e5949.

14. Jiang J, Ballinger CA, Wu Y, Dai Q, Cyr DM, Höhfeld J, Patterson C: **CHIP is a U-box-dependent E3 ubiquitin ligase: identification of Hsc70 as a target for ubiquitylation.** *J Biol Chem* 2001, **276**:42938–44.

15. Ballinger CA, Connell P, Wu Y, Hu Z, Thompson LJ, Yin LY, Patterson C: **Identification of CHIP, a novel tetratricopeptide repeat-containing protein that interacts with heat shock proteins and negatively regulates chaperone functions.** *Mol Cell Biol* 1999, **19**:4535–45.

16. Connell P, Ballinger CA, Jiang J, Wu Y, Thompson LJ, Höhfeld J, Patterson C: **The co-chaperone CHIP regulates protein triage decisions mediated by heat-shock proteins.** *Nat Cell Biol* 2001, **3**:93–6.

17. Hatakeyama S, Matsumoto M, Kamura T, Murayama M, Chui D-H, Planel E, Takahashi R, Nakayama KI, Takashima A: **U-box protein carboxyl terminus of Hsc70-interacting protein (CHIP) mediates poly-ubiquitylation preferentially on four-repeat Tau and is involved in neurodegeneration of tauopathy.** *J Neurochem* 2004, **91**:299–307.

18. Dickey CA, Kamal A, Lundgren K, Klosak N, Bailey RM, Dunmore J, Ash P, Shoraka S, Zlatkovic J, Eckman CB, Patterson C, Dickson DW, Nahman NS, Hutton M, Burrows F, Petrucelli L: **The high-affinity HSP90-CHIP complex recognizes and selectively degrades phosphorylated tau client proteins.** *J Clin Invest* 2007, **117**:648–58.

19. Scaglione KM, Zavodszky E, Todi S V, Patury S, Xu P, Rodríguez-Lebrón E, Fischer S, Konen J, Djarmati A, Peng J, Gestwicki JE, Paulson HL: **Ube2w and ataxin-3 coordinately regulate the ubiquitin ligase CHIP.** *Mol Cell* 2011, **43**:599–612.

20. Todi S V, Scaglione KM, Blount JR, Basrur V, Conlon KP, Pastore A, Elenitoba-Johnson K, Paulson HL: **Activity and cellular functions of the deubiquitinating enzyme and polyglutamine disease protein ataxin-3 are regulated by ubiquitination at lysine 117.** *J Biol Chem* 2010, **285**:39303–13.

21. Todi S V, Winborn BJ, Scaglione KM, Blount JR, Travis SM, Paulson HL: **Ubiquitination directly enhances activity of the deubiquitinating enzyme ataxin-3.** *EMBO J* 2009, **28**:372–82.

22. Tsou W-L, Burr AA, Ouyang M, Blount JR, Scaglione KM, Todi S V: **Ubiquitination regulates the neuroprotective function of the deubiquitinase ataxin-3 in vivo.** *J Biol Chem* 2013, **288**:34460–9.

23. Imai Y, Soda M, Hatakeyama S, Akagi T, Hashikawa T, Nakayama KI, Takahashi R: **CHIP is associated with Parkin, a gene responsible for familial Parkinson’s disease, and enhances its ubiquitin ligase activity.** *Mol Cell* 2002, **10**:55–67.
