# Supplementary material for: STUB1/CHIP mutations cause Gordon Holmes syndrome as part of a widespread multisystemic neurodegeneration: evidence from four novel mutations
Source: Orphanet J Rare Dis. 2017 Feb 13;12:31. doi: 10.1186/s13023-017-0580-x (PMC5307643; doi:10.1186/s13023-017-0580-x)
Supplement: Additional file 2: — Western blots of CHIP in mutation carriers. (DOCX 241 kb) [file 13023_2017_580_MOESM2_ESM.docx]

Additional file 2: Western Blot of CHIP in mutation carriers.

In order to check for possible changes in CHIP protein levels, Western blot analysis was performed using whole cell lysates of fibroblasts from index patients of both families (II.1 family 1; II.4 family 2) compared to fibroblast lines from three control subjects.

*Materials and Methods*

CHIP-mutant and control fibroblasts were harvested and washed once with cold PBS and lysed with RIPA Buffer (SIGMA) including 1x protease inhibitor (cOmplete Mini, Roche). 20 µg proteins were mixed with Pierce^TM^ Lane Marker Reducing Sample Buffer (5x), separated with a 10% Bis-Tris NuPAGE Gel and transferred onto a PVDF membrane (Immobilon, Millipore) over night at 4°C with 25V. For immunoblotting mouse anti-CHIP (G-2) (sc-133066; Santa-Cruz; 1/1000) and mouse anti-GAPDH (H86504M; Meridian; 1/20000) antibodies were used. Membranes were further probed with HRP-coupled secondary anti-mouse antibody (7076; Cell Signaling) and developed with ECL solution (selfmade or Immobilon Western HRP Substrate) and the ChemiDOC MP Imaging System (Bio-Rad).

*Results*

In none of the two STUB1 index patients, a change in protein expression or an expression of a truncated protein was observed. This result was to be expected for the index subject of family 2, where the two *STUB1* *missense* mutations are indeed expected to lead to a functionally impaired protein, rather than a truncated protein. Also in the index subjects of family 1, where the missense variant was combined with a stop variant, the full wild-type protein was still visible. This might be due to the lack of sensitivity of the western blot or by the fact that the protein level is maintained by a compensatory mechanism via the second allele.


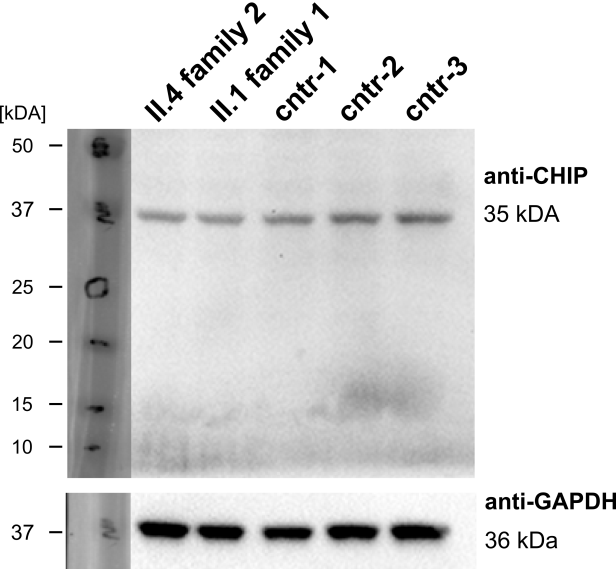


**Figure Additional File 2. Western blot of CHIP in fibroblasts of *STUB1*/CHIP-mutant index patients II.4 (family 2) and II.1 (family 1**) compared to fibroblast lines from three control subjects**.** In none of the two patients, a truncation of the CHIP protein was detected.
