# Supplementary material for: STUB1/CHIP mutations cause Gordon Holmes syndrome as part of a widespread multisystemic neurodegeneration: evidence from four novel mutations
Source: Orphanet J Rare Dis. 2017 Feb 13;12:31. doi: 10.1186/s13023-017-0580-x (PMC5307643; doi:10.1186/s13023-017-0580-x)
Supplement: Additional file 3: — Case vignettes. Detailed medical history and clinical examination data of the three STUB1 patients. (DOCX 14 kb) [file 13023_2017_580_MOESM3_ESM.docx]

Additional file 3

Case vignettes

*Case vignette subject II.1, family 1*

Subject II.1 of family 1 showed a slightly delayed early motor development, with the ability to walk independently at 20 months of age. Between 10 months and 2 years of age, the patient had four generalized seizures. From the age of 2, further motor and cognitive development was unremarkable, the patient played soccer and attended a mainstream school. He underwent surgery because of undescended testes at the age of 7. Hypogonadism was diagnosed at the age of 18, and the patient was subsequently treated with testosterone. Ataxia symptoms started at the age of 12, when the patients’ movements were first noticed as clumsy by his parents. Subsequently his speech became slower. Aged 14, he was diagnosed with ‘ataxia’, and became largely wheelchair bound at the age of 19. Motor and speech function progressively worsened. With 26, the patient was unable to use the wheelchair himself and showed episodes of agitation and aggressive behavior. At the time of admittance to our inpatient unit the 32-year-old patient presented with severe ataxia and severe dementia. Clinical examination revealed a frontal-executive type of dementia with perseverations, verbigerations and loss of orientation concerning location and time, but preserved personal orientation. He was unable to follow simple tasks. The patient had cerebellar dysarthria, saccadic eye movements and a viewing direction nystagmus. He further presented a tremor of trunk and head, and suffered from tetraspasticity with emphasis on the legs as well as a dystonic hand posture. Muscle strength was generally reduced, and reflexes were brisk with broadened reflex zones. The patient was unable to walk or stand by himself. He further suffered from urge incontinence and had a left convex scoliosis.

*Case vignette subject II.1, family 2*

Subject II.1 of family 2 suffered from ataxia starting at the age of 12. He started to use a wheelchair two years later, aged 14, cognitive function was normal at that point. Ataxia progressed and was gradually accompanied by pyramidal tract involvement, the patient was completely wheelchair bound at the age of 21. Severe cognitive deficits were evident at age 23 years. Intellectual and motor abilities further declined, leading to mutism and severe feeding difficulties due to dysphagia, necessitating a gastric tube at age 36. On clinical examination the patient presented with severe dementia resulting in mutism, cerebellar ataxia, intermittent ballistic athetotic movements, increased tendon reflexes and spasticity of the upper and lower limbs. He further had a bilaterally positive Babinki’s sign and ankle clonus, and suffered from urge incontinence. The patient died at the age of 40.

*Case vignette subject II.4, family 2*

His sister, subject II.4 of family 2, had a similar disease course, but disease started with cataract which required surgery at the age of 11. Ataxia and spasticity developed at the age of 20. She briefly worked as a secretary in her early twenties and her intellect was normal until the age of 25, followed by cognitive decline and episodes of paranoid behavior. She needed a wheelchair at the age of 35, and became completely wheelchair bound with 40. At the age of 43 the patient developed feeding difficulties and dysphagia, suffered from complex partial seizures and nearly complete mutism. Clinical examination at the age of 44 revealed a downbeat nystagmus and orofacial dyskinesia, possibly due to the use of neuroleptic medication. She further presented severe titubation and profound tetraspasticity with flexion contractures in all four limbs. Like her brother she presented intermittent ballistic athetotic movements, increased tendon reflexes, a bilaterally positive Babinki’s sign and ankle clonus, as well as urge incontinence from the age of 40.
